# Utilization of AI Among Medical Students and Development of AI Education Platforms in Medical Institutions: Cross-Sectional Study

**DOI:** 10.2196/81652

**Published:** 2026-01-08

**Authors:** Xiaokang Shi, Zewu Jiang, Li Xiong, Ka-Chun Siu, Zhen Chen

**Affiliations:** 1 Renji Hospital, School of Medicine, Shanghai Jiao Tong University, Building 9, 3rd Floor, No. 160, Pujian Road, Pudong New Area, Shanghai, 200127, China, 86 02168383215; 2 School of Medicine, Tongji University, Shanghai, China; 3 Department of Health and Rehabilitation Sciences, College of Allied Health Professions, University of Nebraska Medical Center, Omaha, NE, United States

**Keywords:** AI chatbots, artificial intelligence, cross-sectional study, medical education, medical schools, medical students, technology acceptance

## Abstract

**Background:**

The emergence of artificial intelligence (AI) is driving digital transformation and reshaping medical education in China. Numerous medical schools and institutions are actively implementing AI tools for case-based learning, literature analysis, and lecture support. This expanding application is accelerating the adoption of localized AI platforms, which are poised to become integral components in the coming years.

**Objective:**

The primary aim of this study was to investigate the current use of AI tools among medical students, including usage frequency, commonly used platforms, and purposes of use. The second aim was to explore students’ needs and expectations toward AI-powered medical education platforms by collecting and assessing student feedback, and to identify practical requirements across disciplines and academic stages to inform more effective platform design.

**Methods:**

Based on the task-technology fit model and 5 hypotheses, an anonymous online questionnaire was conducted to assess AI usage in learning, gather student feedback on AI-powered medical education platforms, and evaluate expected functionalities. The survey was conducted from March 1 to May 31, 2025, using a convenience sampling method to recruit medical students from various disciplines across Shanghai, China. The sample size was determined at 422, accounting for a 10% rate of invalid responses. The questionnaire was developed and distributed online via Wenjuanxing and promoted through WeChat groups and in-person interviews. Data analysis was conducted employing IBM SPSS Statistics (v 27.0).

**Results:**

A total of 428 valid questionnaires were collected. The average frequency of AI-assisted learning among medical students was 5.06 (SD 2.05) times per week. Over 90% (388/428) of the students used more than 2 AI tools in their daily tasks. Students from different disciplines, educational stages, and academic systems demonstrated different usage patterns and expectations for AI-powered medical education platforms.

**Conclusions:**

AI technology is widely accepted by medical students and is extensively applied across various aspects of medical education. Significant differences are observed in usage patterns across disciplines, educational stages, and academic systems. Understanding the actual needs of students is crucial for the construction of AI-powered medical education platforms.

## Introduction

The rapid development of artificial intelligence (AI) has profoundly accelerated the digital transformation of medical education worldwide. AI demonstrates significant potential across multiple domains of medical education, including case-based teaching, literature analysis, and lecture support [1,2].

Globally, previous studies have documented the successful integration of large language models (LLMs) and conversational agents in medical education, significantly enhancing teaching effectiveness [3,4]. Gilson et al [5] reported that ChatGPT and other LLMs have been deployed to simulate clinical reasoning sessions, automate feedback on student essays, and generate customized practice questions, demonstrating measurable gains in diagnostic accuracy and pedagogical efficiency. Simultaneously, students’ mastery of both theoretical knowledge and practical skills has markedly improved, resulting in better learning outcomes and clinical performance. These advances address existing challenges in medical education and open up promising pathways for its future development [6,7].

In China, supportive national policies and rapid technological advances have jointly facilitated the localized application of AI in medical education [8]. The national “AI Plus” Implementation Guidelines outline the strategic direction for the deep integration of AI technologies with public welfare services [9]. Meanwhile, Shanghai’s pioneering Medical Artificial Intelligence Work Plan specifically proposes establishing an “intelligent medical education and training” platform, emphasizing the development of smart training environments using generative AI technologies [10]. This provides clear policy support and practical guidance for higher education institutions to develop localized AI−powered medical education platforms [11,12].

With the widespread adoption of AI, students’ learning methods, habits, and institutional teaching models have been rapidly reshaped. However, systematic data on medical students’ current AI usage patterns and their practical needs across different educational stages and disciplines remain limited. A deeper understanding of these aspects is crucial for effectively guiding the development and optimization of future AI−powered medical education platforms. Therefore, this study aimed to collect medical students’ current use of AI and their practical needs across different educational stages and academic disciplines. We also explored effective strategies for developing AI-powered medical education platforms, with the goal of providing recommendations to guide the development and optimization of future AI−powered medical education platforms.

## Methods

### Participants and Procedures

This study employed a convenience sampling method to conduct a cross-sectional survey among medical students from various medical universities in Shanghai, China, with data collected from March 1 to May 31, 2025.

The inclusion criteria were (1) current enrollment in a medical program and (2) provision of informed consent to participate. The exclusion criteria were (1) nonmedical students, (2) students not currently enrolled in any program, (3) students attending medical schools outside Shanghai, and (4) students who declined participation.

### Theoretical Framework and Hypotheses

This study is based on the task-technology fit (TTF) model. Its core proposition is that technology’s effectiveness is determined not by its attributes or user attitudes alone but by the fit between technological functionalities and user task requirements.

Based on the TTF framework and a review of relevant literature [13,14], this study proposes 5 hypotheses. These hypotheses aim to examine differences in usage status, functional needs, and expectations regarding AI-powered medical education platforms among medical students across different disciplines, educational stages, and program types while identifying factors with a significant influence.

- H1: Perceived task-technology fit has a significant positive impact on platform satisfaction.
- H2: Disciplinary background moderates the relationship between TTF and platform satisfaction.
- H3: Program type (full-time vs part-time) moderates the relationship between TTF and platform satisfaction.
- H4: Educational stage moderates the relationship between TTF and platform satisfaction.
- H5: The frequency of AI usage has a significant positive impact on platform satisfaction.

### Instrument Pretesting and Validation

Guided by these 5 hypotheses, we designed a concise online questionnaire. Prior to the formal survey, a pilot test was conducted through in-person interviews with 23 postgraduate clinical medicine students from the same institution to evaluate content validity, item clarity, and internal consistency reliability. The expected platform functions scale (Cronbach *α*=0.825, items with zero variance were excluded) and the most frequently used AI platforms scale (Cronbach *α*=0.858, items with zero variance were excluded) showed high internal consistency.

### Questionnaire Design

Based on the feedback from the pilot study, we made appropriate revisions to certain items and their phrasing in the questionnaire. Our questionnaire covered 3 sections with 13 items (see the questionnaire in Multimedia Appendix 1): the first section is general information, including age, gender, major, school, educational stage, and academic program type; the second section is the current use of AI tools, including the frequency of use (average per week), preferred platforms, and usage purposes (eg, theoretical learning, literature assistance, among others); and the third section is current status and expectations for AI-powered medical education platforms (whether the institution has developed an AI-powered medical education platform, satisfaction with the platform, and expected functions of the future platform) [13].

To ensure data completeness, all questionnaire items were set as mandatory, and participants were required to complete all the questions before submission. The survey platform would automatically record device type and completion time.

### Ethical Considerations

This study received an ethics exemption (EX-2025‐017) from the Medical Ethics Committee of Renji Hospital, Shanghai Jiao Tong University School of Medicine, as it utilizes anonymized data, operates under standard informed consent protocols, and involves no sensitive biological materials or procedures.

We obtained informed consent from all student participants before the survey, providing full details about the study’s purpose, procedures, and privacy protections. No compensation was provided, as the study involved minimal burden and no anticipated harm. All the data were strictly protected to ensure confidentiality and prevent any risk of information leakage. To this end, access was restricted to authorized research team members, and the data were used solely for analysis and reporting within this study.

### Sample Size

To ensure adequate statistical power and precision for the intended analyses, we used a standard sample size calculation formula [15]. Assuming a 95% CI, a margin of error of 0.05, and an expected population proportion of 0.5, the minimum required sample size was calculated to be 384. Drawing on previous studies [16,17] and to improve the generalizability of the results, we further accounted for a 10% invalid questionnaire rate, resulting in a final target sample size of 422.

$n=\frac{Z²\cdot P\cdot \left(1-P\right)}{E²}$ (95% CI, *Z*=1.96, *E*=0.05, *P*=.50)

Finite population correction was not applied due to the use of convenience sampling and structural heterogeneity across institutions, which precluded the definition of a single unified sampling frame.

### Statistical Analysis

Statistical analyses were performed using IBM SPSS Statistics (version 27.0). Descriptive statistics were computed using appropriate measures for each variable type: continuous variables were summarized with means and SDs, while categorical variables were presented as frequencies and percentages.

For group comparisons involving categorical variables, chi-square tests were employed. Multiple response analyses were conducted using multiple response sets combined with chi-square tests, with Bonferroni correction applied to account for multiple comparisons. For ordinal data or continuous variables violating normality assumptions, non-parametric tests (Mann-Whitney *U* for 2-group comparisons and Kruskal-Wallis *H* for multigroup comparisons) were utilized, with post hoc analyses performed where appropriate.

ANOVA was used for comparing continuous variables across multiple groups, while MANOVA was employed for analyses involving multiple continuous dependent variables. Multivariable analyses included linear regression for continuous outcomes and logistic regression for binary outcomes.

All statistical tests used a 2-tailed significance threshold of *P*<.05, with appropriate corrections for multiple testing implemented where necessary [18].

## Results

### Participants’ Characteristics

A total of 440 questionnaires were collected. After excluding 12 responses from nonmedical students, students at institutions outside Shanghai, and nonenrolled individuals, 428 valid questionnaires were retained, yielding an effective response rate of 97.3%. The questionnaires were returned with complete and valid responses, thus containing no missing data.

Participants were drawn from 7 medical schools in Shanghai, with 188 (43.92%) male participants and 240 (56.07%) female participants, and a median age of 22 (IQR 20.07-24.62) years. Among them, 223 (52.10%) were undergraduate students, 174 (40.65%) were master’s students, and 31 (7.24%) were doctoral students. The sample covered 8 major disciplines: clinical medicine, basic medicine, rehabilitation therapy, nursing, public health and epidemiology, pharmacy, traditional Chinese medicine, and medical engineering.

### Frequency of AI Use Among Medical Students

Most respondents reported regular use of AI tools in academic work and daily tasks, with a mean usage frequency of 5.06 (SD 2.05) times per week. The mean (SD) frequency among undergraduates, master’s students, and doctoral students was 5.09 (1.97), 4.99 (2.11), and 5.19 (2.27) times per week, respectively, with no significant differences based on educational stage or gender (Table 1).

**Table 1.** Current usage of artificial intelligence (AI) in medical students’ learning

Variables	Educational stage			Chi-square (*df*)	Cramer *V*	*P* value^a^	Gender		Chi-square (*df*)	Cramer *V*	*P* value^a^	Total (n=428), n (%)
	Undergraduate students (n=223), n (%)	Master’s students (n=174), n (%)	Doctoral students (n=31), n (%)				Male (n=188), n (%)	Female (n=240), n (%)				
Average weekly use of AI for learning				7.97 (6)	0.10	.24			6.29 (3)	0.12	.10	
≤1	15 (6.73)	21 (12.07)	4 (12.90)				16 (8.51)	24 (10)				40 (9.35)
2‐3	58 (26.01)	34 (19.54)	6 (19.35)				36 (19.15)	62 (25.83)				98 (22.90)
4‐6	52 (23.32)	44 (25.29)	4 (12.90)				40 (21.28)	60 (25.00)				100 (23.36)
≥7	98 (43.95)	75 (43.10)	17 (54.84)				96 (51.06)	94 (39.17)				190 (44.39)
AI platform used by medical students												
DeepSeek	213 (95.52)	163 (93.68)	28 (90.32)	1.67 (2)	0.06	.43	176 (93.62)	228 (95.00)	0.38 (1)	0.03	.54	404 (94.39)
Doubao	138 (61.88)	99 (56.90)	13 (41.94)	4.74 (2)	0.11	.09	97 (51.60)	153 (63.75)	6.41 (1)	0.12	.01	250 (58.41)
Kimi	127 (56.95)	108 (62.07)	13 (41.94)	4.57 (2)	0.10	.10	98 (52.13)	150 (62.50)	4.66 (1)	0.10	.03	248 (57.94)
ChatGPT	156 (69.96)	103 (59.20)	21 (67.74)	5.08 (2)	0.11	.08	130 (69.15)	150 (62.50)	2.06 (1)	0.07	.15	280 (65.42)
ChatGLM	28 (12.56)	14 (8.05)	0 (0)	5.88 (2)	0.12	.05	17 (9.04)	25 (10.42)	0.23 (1)	0.02	.64	42 (9.81)
Claude	24 (10.76)	9 (5.17)	3 (9.68)	4.03 (2)	0.10	.13	22 (11.70)	14 (5.83)	4.71 (1)	0.11	.03	36 (8.41)
Gemini	29 (13.00)	13 (7.47)	0 (0)	7.02 (2)	0.13	.03	27 (14.36)	15 (6.25)	7.84 (1)	0.14	.005	42 (9.81)
Other^b^	22 (9.87)	11 (6.32)	1 (3.23)	2.70 (2)	0.08	.26	16 (8.51)	18 (7.50)	0.15 (1)	0.02	.70	34 (7.94)
Number of AI platforms used by medical students				17.36 (14)	0.14	.24			3.86 (7)	0.10	.80	
2	53 (23.77)	45 (25.86)	8 (25.81)				46 (24.47)	60 (25.00)				106 (24.77)
≥2	210 (94.17)	153 (87.93)	25 (80.65)				166 (88.30)	222 (92.50)				388 (90.65)
≥3	157 (70.40)	108 (62.07)	17 (54.83)				120 (63.83)	162 (67.50)				282 (65.89)

a Chi-square test and Bonferroni correction were applied for multiple comparisons (*α*=.00625).

b AI platforms, including Qwen Chat, ERNIE Bot, Tencent Yuanbao, Poe, and Grok, were grouped together for analysis due to their relatively small sample sizes.

### AI Platforms Used by Medical Students

In terms of AI platform selection, most medical students favor mainstream tools such as DeepSeek (n=404, 94.39%), Doubao (n=250, 58.41%), and ChatGPT (n=280, 65.42%) for their daily study and work. Survey results further indicated that the current use of multiple AI platforms has become a common practice among medical students, with over 90% (388/428) of the students reporting the use of 2 or more platforms, and over 60% (282/428) of the students reported using 3 or more platforms (Table 1).

Gender-based differences were observed in the adoption of specific AI platforms, with selected variations reaching statistical significance (*χ*²_1_*=*7.84; *P*=.005). Nonetheless, the overall variety of the platforms utilized remained consistent across genders, as evidenced by a comparable number of the tools used (*χ*²_7_*=*3.86; *P*=.80).

### AI Usage Patterns by Academic Program Type and Educational Stage

The application of AI in medical education spans multiple areas, including theoretical learning, question analysis, literature translation, and scientific research. Based on the literature review and preliminary survey feedback, our questionnaire categorized the application areas into 5 domains: “theoretical learning,” “exam question analysis,” “information retrieval,” “literature interpretation,” and “research design and data analysis.”

**Figure 1.**
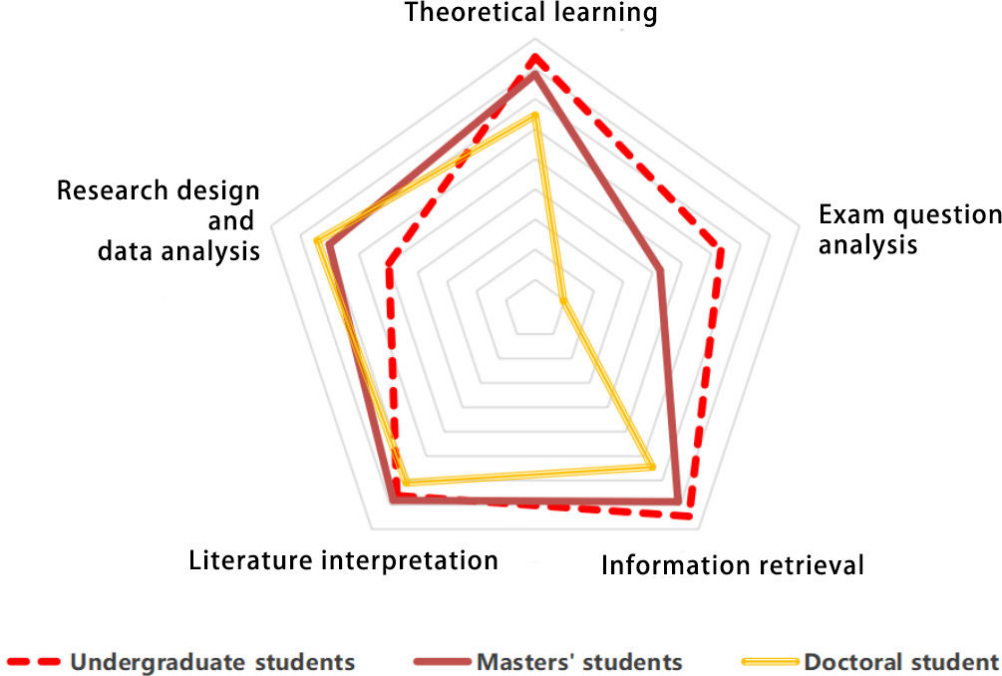
Usage of artificial intelligence (AI) tools in learning among medical students from different educational stages.

Regarding educational stage, undergraduate students primarily used AI for exam preparation, while graduate students focused more on research tasks such as study design and data analysis (Figure 1).

**Figure 2.**
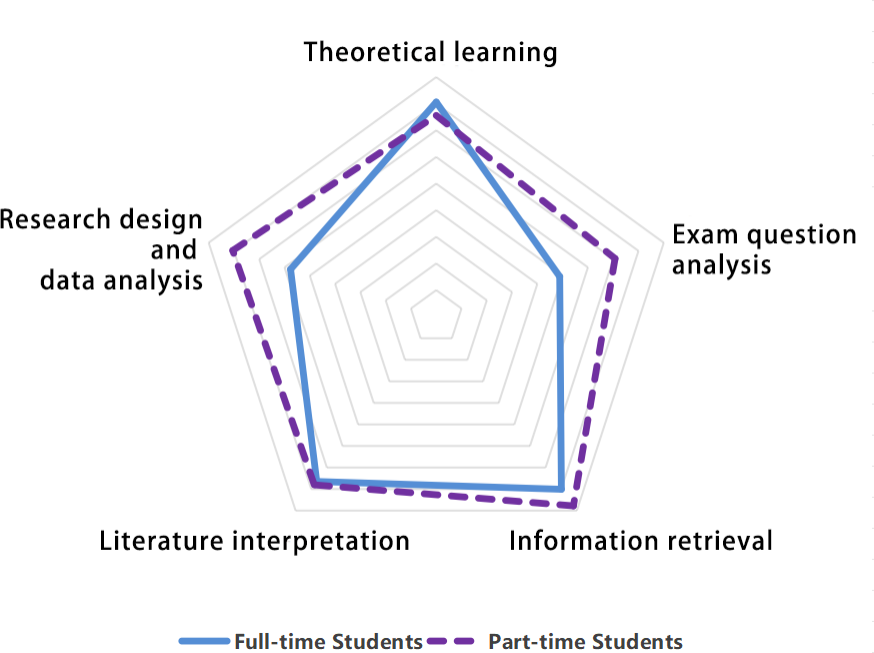
Usage of artificial intelligence (AI) tools in learning among medical students from different academic program types (full-time vs part-time).

When grouped by academic program type, part-time students demonstrated stronger needs for AI support in practical research and exam question analysis. This preference pattern appears related to their need to balance studies with work commitments, requiring efficient learning solutions that yield immediate academic and professional applicability (Figure 2).

### AI Usage Patterns by Disciplines

Medical students from different academic disciplines exhibited distinct priorities in their application of AI-powered learning (Table 2). Initial analysis using a multiple response test revealed a significant overall difference in AI usage patterns across disciplines (*χ*²_30_=53.62; *P*=.005), prompting subsequent pairwise comparisons between disciplines with a Bonferroni-adjusted alpha of 0.01.

**Table 2.** Comparative analysis of artificial intelligence (AI) function usage across different medical disciplines.

Domain	Clinical medicine (n=245), n (%)	Nursing (n=65), n (%)	Rehabilitation therapy (n=31), n (%)	Basic medicine (n=31), n (%)	Public health and epidemiology and pharmacy^a^ (n=11), n (%)	Traditional Chinese medicine (n=22), n (%)	Medical engineering (n=23), n (%)	Chi-square (*df*)	Cramer *V*	*P* value^b^
Theoretical learning	201 (82.04)	50 (76.92)	24 (77.42)	26 (83.87)	8 (72.73)	16 (72.73)	18 (78.26)	2.58 (6)	0.08	.86
Exam question analysis	139 (56.73)	37 (56.92)	12 (38.71)	14 (45.16)	4 (36.36)	7 (31.82)	5 (21.74)	18.49 (6)	0.21	.005
Information retrieval	202 (82.45)	56 (86.15)	19 (61.29)	28 (90.32)	8 (72.73)	15 (68.18)	18 (78.26)	13.89 (6)	0.18	.03
Literature interpretation	184 (75.10)	55 (84.62)	22 (70.97)	26 (83.87)	10 (90.91)	12 (54.55)	19 (82.61)	11.80 (6)	0.17	.07
Research design and data analysis	144 (58.78)	35 (53.85)	17 (54.84)	23 (74.19)	9 (81.82)	15 (68.18)	13 (56.52)	7.02 (6)	0.13	.32

a Disciplines including public health and epidemiology and pharmacy were grouped together for analysis due to their relatively small sample sizes.

b Chi-squared test and Bonferroni correction were applied for multiple comparisons (*α*=.01).

Across all disciplines, the usage demand for theoretical learning was similarly high, with no statistically significant differences identified.

Regarding exam question analysis, students in clinical medicine demonstrated significantly higher usage demand than those in medical engineering (*χ*²_1_=10.36; *P*=.001).

In terms of information retrieval, students from rehabilitation therapy demonstrated significantly lower usage demand compared to students in clinical medicine (*χ*²_1_=7.72; *P*=.005), nursing (*χ*²_1_=7.59; *P*=.006), and basic medicine (*χ*²_1_=7.12; *P*=.008).

For literature interpretation, traditional Chinese medicine students demonstrated a significantly lower level of usage demand relative to students in nursing (*χ*²_1_=8.40; *P=*.004).

Across disciplines, usage demand for research design and data analysis was moderate, peaking non-significantly among public health and epidemiology and pharmacy students.

### Correlates of Satisfaction With the Institutional AI Platform

With the rapid development of AI, many universities have launched localized AI platforms. Our survey investigated the availability of institution-specific AI−powered medical education platforms among medical students. Furthermore, we assessed student satisfaction with these platforms.

Approximately one-fifth (86/428) of the respondents reported that their institutions had developed such platforms. Satisfaction scores among these users exhibited significant variation. The average satisfaction score among the 86 users was 72.23 (SD 21.84), distributed as 40 (46.51%) satisfied, 28 (32.56%) neutral, and 18 (20.93%) dissatisfied.

Nonparametric tests revealed a significant difference in satisfaction levels by gender (*U*=686.50; *z*=−2.06; *P*=.04). No significant associations were found with academic program type, educational stage, or discipline. A subsequent multivariable regression that included these variables and usage frequency identified no significant predictors. To assess the model’s reliability, collinearity diagnostics were performed, and they revealed no substantial multicollinearity (Table 3).

**Table 3.** Medical students’ satisfaction with the artificial intelligence (AI)−powered medical education platforms at their institutions.

Predictor	Unstandardized coefficient		Standardized coefficient	*P* value	VIF^a^	95% CI for unstandardized coefficients (*B*)
	*B*	SE	*β*			
Constant	71.97	13.93	—^b^	<.001	—	44.22 to 99.72
Academic program type (reference: part-time)						
Full-time	−3.18	9.72	−0.04	.74	1.20	−22.55 to 16.19
Gender (reference: female)						
Male	8.52	5.12	0.20	.10	1.11	−1.68 to 18.72
Frequency	−0.39	1.34	−0.03	.77	1.08	−3.06 to 2.28
Discipline (reference: clinical medicine)						
Nursing	5.59	6.85	0.10	.42	1.14	−8.05 to 19.24
Rehabilitation therapy	−4.14	13.76	−0.04	.76	1.08	−31.55 to 23.26
Basic medicine	−0.36	7.38	−0.01	.96	1.11	−15.07 to 14.35
Public health and epidemiology and pharmacy	10.54	12.64	0.10	.41	1.20	−14.64 to 35.71
Medical engineering	−6.18	24.99	−0.03	.81	1.22	−55.96 to 43.61
Educational stage (reference: undergraduate student)						
Master’s student	−2.43	5.97	−0.05	.69	1.33	−14.32 to 9.46
Doctoral student	8.59	10.83	0.10	.43	1.29	−12.99 to 30.18

a VIF: variance inflation factor.

b Not applicable.

The lack of significant predictors for satisfaction should be interpreted with caution. This result may reflect the inherently subjective and multifaceted nature of satisfaction, which can be influenced by factors beyond the scope of this study. Future research employing longitudinal or mixed methods designs is needed to unravel the complex drivers of user satisfaction.

### Correlates of Expected Functions for the Institutional AI Platform

The majority (342/428) of the students reported that their institutions had not yet launched an AI platform specifically centered on medical education. To investigate the needs of this group, this survey further explored their practical needs and expectations for upcoming platforms.

Drawing on the current practical applications of AI technology and feedback from medical students, along with a review of relevant literature, our questionnaire categorized the expected functions of AI-powered medical education platforms into 8 aspects: literature translation and interpretation, exam question analysis, clinical trial assistance, basic laboratory support, knowledge mapping, virtual simulation platforms, frontier knowledge navigation, and intelligent emotional support.

**Figure 3.**
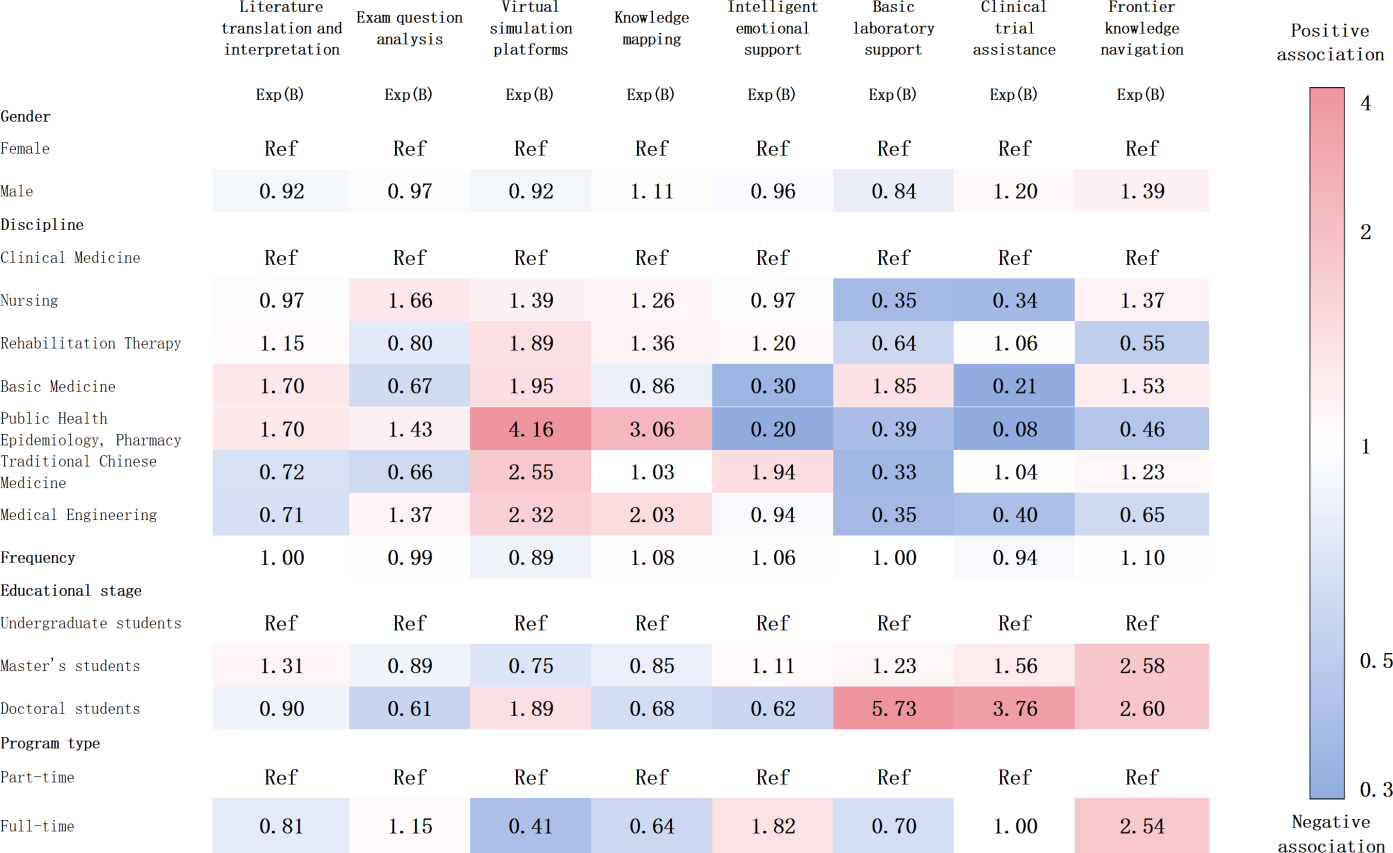
Expected functions for artificial intelligence (AI) medical education platforms across student subgroups.

Nonparametric testing revealed significant subgroup differences in the demand for specific platform functions. Guided by these initial findings, we advanced the analysis using binary logistic regression within a more rigorous multivariable framework. For comparing the relative effects of multiple predictors, the results are presented as odds ratios (Exp(B)) and visualized in the accompanying heatmap (Figure 3).

Regarding gender, the regression analysis revealed a consistent pattern: no statistically significant associations were found between gender and any of the anticipated platform functions.

Analysis of academic program type (full-time vs part-time) revealed no significant differences in functional expectations. However, full-time students demonstrated a numerically stronger preference for frontier knowledge navigation (*B*=0.93; Exp(B)=2.54; *P*=.14), while they were less inclined toward Virtual Simulation Platforms (*B*=−0.90; Exp(B)=0.41; *P*=.08).

Regarding the educational stage, doctoral students demonstrated a significantly stronger preference for AI-assisted clinical trial support (*B*=1.32; Exp(B)=3.76; *P*=.01) and basic laboratory assistance (*B*=1.75; Exp(B)=5.73; *P=*.001) compared to undergraduates, while master’s students showed significantly higher demand for frontier knowledge navigation (*B*=0.95; Exp(B)=2.58; *P=*.02).

Across different academic disciplines, medical students showed varying expectations for the functions of upcoming AI-powered medical education platforms. Students in clinical medicine demonstrated a significantly stronger demand for clinical trial assistance (*B*=−1.09; Exp(B)=0.34; *P*=.008) and Basic Laboratory Support (*B*=−1.05; Exp(B)=0.35; *P*=.005) compared to those in nursing. Conversely, nursing students were inclined toward personalized guidance on theoretical exam preparation, though this tendency did not reach statistical significance (*B*=0.51; Exp(B)=1.66; *P=.*08). Notably, students in clinical medicine demonstrated a significantly higher demand for clinical trial assistance compared to students in other disciplines. This difference reached statistical significance when compared specifically with students in basic medicine (*B*=−1.58; Exp(B)=0.21; *P*=.01) and those in public health and epidemiology and pharmacy (*B*=−2.54; Exp(B)=0.08; *P*=.02).

### Test of the TTF Hypotheses

Based on the results of our prior data analysis and guided by the 5 research hypotheses derived from the TTF theory, we employed appropriate statistical methods to test these hypotheses (Table 4).

**Table 4.** Hypothesis testing results of the task-technology fit (TTF) model.

Hypothesis	Path relationship	Statistic^a^	Effect size^b^	*P* value	Supported
H1	TTF (current use) → satisfaction	*F*(5, 80)=1.11	Adjusted *R*²=0.006	.36	No
H2	Discipline → TTF (current use)	*F*(36, 2526)=1.60	Partial η²=0.02	.01	Yes
	Discipline → TTF (expected function)	*F*(42, 840)=1.25	Partial η²=0.06	.13	No
H3	Academic program type → TTF (current use)	*F*(6, 421)=3.32	Partial η²=0.05	.003	Yes
	Academic program type → TTF (expected function)	*F*(7, 140)=1.22	Partial η²=0.06	.29	No
H4	Educational stage → TTF (current use)	*F*(12, 842)=6.51	Partial η²=0.09	*<*.001	Yes
	Educational stage → TTF (expected function)	*F*(14, 280)=1.53	Partial η²=0.07	.10	No
H5	Frequency → satisfaction	*B*=−0.39	95% CI for *B*=−3.06 to 2.28	.77	No

a Analytical methods were hypothesis-specific: ANOVA for a single outcome variable (H1); multivariate ANOVA for multiple outcome variables (H2-H4); linear regression with unstandardized coefficients for predictive modeling (H5).

b Effect size measures were selected and reported in accordance with the conventions for the specific statistical procedures applied.

The analytical approach was tailored to the characteristics of the variables under examination. For H1, ANOVA was used, which indicated no significant association between current AI usage patterns and satisfaction. For H2, H3, and H4, MANOVA was applied: the results showed that discipline, academic program type, and educational stage each had a significant influence on TTF (current use); however, none of these factors demonstrated a significant effect on TTF (expected function). For H5, linear regression analysis found no significant relationship between frequency and satisfaction.

## Discussion

While previous studies have described broad trends in AI adoption among medical students, this study identifies the specific drivers of heterogeneity within a defined cohort from Shanghai. Through a granular subgroup analysis, we demonstrate how disciplinary background, educational stage, and program type significantly shape distinct patterns in usage frequency, functional preferences, and perceived value.

### Popularity of AI Technology Among Medical Students

The rapid advancement of AI technology is demonstrating the potential to reshape traditional paradigms in medical education. For medical students, the integration of digital and intelligent technologies has significantly enhanced instructional quality and learning outcomes. With ongoing technological advancement, AI is now widely adopted and has become an essential part of students’ academic work [19].

The survey revealed that medical students now engage with AI tools on a frequent basis, reflecting a notable shift in usage patterns compared to earlier adoption phases [20,21]. This suggests that, with ongoing technological advancement and the growing accessibility of AI, students are increasingly adopting this new technology and using it more in their learning.

In terms of LLM selection, most students preferred mainstream models, encompassing both domestic platforms such as DeepSeek and Doubao and international ones like ChatGPT. The choice appears to have been influenced by factors such as accessibility and performance. While mainstream LLMs meet most students’ academic requirements, many still select specific models based on personal preferences and practical needs. This reflects a growing trend toward individualized AI tool usage.

Regarding mainstream LLM selection, international findings show that ChatGPT is widely popular globally [22,23]; however, its adoption among the surveyed medical students in Shanghai remains lower compared to DeepSeek. This observed usage pattern aligns with DeepSeek’s established localization strategy and regulatory compliance within the Chinese environment. China’s regulatory policies require GenAI services to complete local filing and security assessments [24]. ChatGPT, lacking such compliance, is inaccessible through conventional channels. Furthermore, China’s exclusion from OpenAI’s supported countries creates additional access barriers [25]. In contrast, DeepSeek operates in full compliance with these requirements, ensuring seamless accessibility for Chinese users and thus gaining a competitive edge in the local AI market.

The analysis of AI tool preferences revealed distinct gender−based patterns. Despite using a similar number of AI platforms, with mainstream tools being central for both groups, male students demonstrated greater enthusiasm for emerging options, whereas female students adopted a more cautious approach. These observed differences align with previous findings on gendered perceptions of AI technology [26-28]. Notably, this variation further underscores the importance of developing flexible, multiplatform strategies to accommodate diverse user preferences in medical education.

The integration of AI technology has become a defining feature of contemporary medical education. Our findings reveal a clear consensus among students across all academic backgrounds on adopting a multiplatform approach. Regardless of educational stage or discipline, medical students are actively leveraging diverse AI tools, flexibly selecting platforms according to specific learning scenarios and practical needs [29].

### Group Differences in AI Tool Usage for Academic Purposes

Medical knowledge covers a broad spectrum and involves extensive interdisciplinary integration. Although various medical specialties are inherently connected, they differ significantly in teaching method, clinical practice, and scientific research. These distinctions are also reflected in the application of AI.

From an educational stage perspective, undergraduate students tended to emphasize AI-assisted analysis of theoretical exam questions more than master’s and doctoral students, who placed greater focus on AI’s role in supporting practical research. This difference reflects the distinct teaching priorities at each educational stage: undergraduate students face a heavy burden of theoretical courses and professional qualification exams, so their learning focus tends to be on theoretical exam question analysis and knowledge mapping. In contrast, graduate students, facing less exam pressure, focus more on research-related tasks and thus place greater emphasis on AI as a tool for practical research support [30-32].

From the perspective of academic program type (full-time vs part-time), part-time students had broader and more comprehensive needs for AI-assisted learning compared to full-time students. Their demand for AI in scientific research and test analysis was more pronounced. This stems directly from the dual pressures faced by part-time medical students, who balance both academic studies and daily work. In dealing with research and exams, they tend to be more reliant on AI for assistance and guidance to alleviate the burden of learning [33].

From a disciplinary perspective, different majors have a significant impact on the direction of AI-assisted learning [34]. The data showed that students majoring in clinical medicine and nursing were more focused on AI-assisted exam question analysis [35], while traditional Chinese medicine students had a notably lower demand for literature translation and interpretation. Students in basic medicine, public health and epidemiology, and pharmacy were more concerned with AI’s role in supporting research work. These differences directly reflect the distinct curricular focus, professional requirements, and learning objectives characterizing each discipline [7,20,36].

### Student-Centered Design of Platform Functions

At present, the application of AI in medical education is still in an exploratory stage. Major computer and internet companies, both domestically and internationally, have launched AI models with diverse functionalities. In parallel, universities and research institutions have adopted various strategies to expand the potential of this emerging field. For example, the AI application platform at Tongji University integrates multiple large models such as DeepSeek, OpenAI, and Tongyi Qianwen, offering a wide range of AI tools. Faculty and students can select these tools based on their professional and academic requirements [37]. Similarly, Shanghai Jiao Tong University has launched the “Jiao Xiao Zhi” agent management platform, integrating LLMs including DeepSeek for localized AI deployment. The platform enables faculty and students to create customized AI assistants, streamlining teaching and research tasks through its secure campus−based infrastructure [38].

This survey focused on medical students’ satisfaction with and expectations for AI-powered medical education platforms. While overall satisfaction was relatively high, a significant minority (approximately one-quarter) expressed dissatisfaction, with notable variations across academic stages and disciplines.

To address these divergent needs, we propose a tiered design framework for future platforms:

For undergraduate students: Platforms should prioritize structured learning support. This includes AI-powered tools for adaptive test preparation aligned with standardized exams, interactive virtual patient cases for foundational clinical exposure, and personalized review systems that target individual knowledge gaps.

For postgraduate students (master’s or PhD): The focus should shift to research and specialized skill development. Key features should encompass advanced literature interpretation aids, data analysis modules for processing experimental or clinical data, and AI assistants for research design and grant writing, catering to their deep engagement in academic research.

Discipline-specific customization: Further refinement should distinguish between clinical and basic medicine disciplines. Clinically oriented tracks would benefit from advanced diagnostic simulators and patient management tools, while basic medicine tracks require robust support for experimental design, genomic data analysis, and scientific visualization.

By moving beyond a one-size-fits-all model to adopt such a stratified and discipline-aware approach, AI platforms can achieve deeper integration into medical education, ultimately enhancing both student satisfaction and educational outcomes [9,39].

### Limitations

Due to constraints in personnel and resources, this study employed a convenience sampling method among medical students in Shanghai. This nonprobability sampling approach may have introduced selection bias, such as an overrepresentation of students already enthusiastic about AI, and limits the generalizability of the findings beyond similar urban, well-resourced contexts.

Moreover, this study relied on self-reported measures for key metrics—including AI usage frequency and satisfaction—for which formal psychometric validation was not conducted. This approach increased susceptibility to recall and social desirability biases across all these measures. Furthermore, as subjective constructs, they were vulnerable to varying personal interpretations and benchmarks. This was particularly relevant for abstract constructs like satisfaction. Consequently, these subjective data may not fully capture the nuanced realities of actual user behavior.

Future research could therefore prioritize multicenter, large-sample designs that utilize standardized instruments. Such approaches would provide more robust and generalizable evidence to effectively guide the development of AI-powered medical education platforms.

### Conclusions

This study conducted a cross-sectional survey across major medical institutions in Shanghai, China, collecting and analyzing the current use and practical needs of AI among medical students from different educational stages, academic program types, and disciplines. Our findings clearly indicate that AI is widely applied in medical education and has become a common tool for student learning. Students from different disciplines, educational stages, genders, and academic program types show significant differences in their functional demands for AI-assisted learning.

Furthermore, our study investigated the current status of AI-powered medical education platforms and explored students’ expectations for such platforms. It is evident that the rapid integration of AI in medical education holds great promise, and our findings provide evidence-based support to guide the future development of AI-powered medical education platforms.

## Supplementary material

10.2196/81652 Multimedia Appendix 1 Detailed items of our questionnaire.

10.2196/81652 Checklist 1 CHERRIES checklist.

## Abbreviations

AI: artificial intelligence
LLM: large language model
TTF: task-technology fit model
