# Supplementary material for: Utilization of AI Among Medical Students and Development of AI Education Platforms in Medical Institutions: Cross-Sectional Study
Source: JMIR Hum Factors. 2026 Jan 8;13:e81652. doi: 10.2196/81652 (PMC12782625; doi:10.2196/81652)
Supplement: Multimedia Appendix 1 [file humanfactors-v13-e81652-s001.docx]

**Supplementary File**

**Appendix 1 Questionnaire Design**

**Questionnaire: Status and Prospects of AI-Powered Medical Education Platforms**

Thank you for participating in this survey on AI-powered medical education. The survey will take approximately 3-5 minutes to complete. This questionnaire aims to understand the current status, functional expectations, and user perspectives regarding AI applications in medical education.

This study has been granted an exemption from ethics approval. Your participation is anonymous, and all data collected will remain strictly confidential, used solely for academic research purposes. By proceeding with the survey, you provide informed consent to participate. You may withdraw at any time without consequence.

Please respond based on your actual experiences and perspectives. If you have any questions related to this research, you can contact the researchers of this project.

**Part One General Information**

1. Your gender: _______ **[Single choice question] ***

□Male

□Female

1. Please select the age group you belong to:________**[Single choice question] ***

□18-21

□22-25

□26-29

□30+

1. Name of your institution: _________________ **[Fill in the blank] ***
2. Your major: _________________ **[Fill in the blank] ***
3. Your educational stage:______________ **[Single choice question] ***

□Undergraduate student

□Master’s student

□Doctoral student

1. Your academic program type:__________ **[Single choice question] ***

□Full-time

□Part-time

**Part Two**  **Current Use of AI Tools**

1. Approximately how frequently do you use AI in your daily study, work, and life? **[Single choice question] ***

□Once per week or less on average

□Up to 3 times per week on average

□More than 3 times per week on average

□Used daily on average

1. Which of the following AI large language models have you used? Please select all that apply, and then rank the ones you use from most frequently to least. **[Multiple-choice ranking question]***

□DeepSeek

□Doubao

□Kimi

□ChatGPT

□ChatGLM

□Claude

□Gemini

□Other (Please fill in the blank: __________ )

1. How do you apply AI in your medical studies? Please select all that apply. **[Multiple choice question]***

□Theoretical Learning

□Exam Question Analysis

□Information Retrieval

□Literature Interpretation

□Research Design and Data Analysis

□Other (Please fill in the blank: __________ )

**Part Three** **Construction of AI-powered medical education platform**

1. Has your medical school launched a proprietary AI-powered medical education platform? **[Single choice question] ***

□Yes

□No

□Not sure

1. **(Depends on the first option of question 10)** How satisfied are you with the AI-powered medical education platform provided by your school? **[score] ***

**(0=Unsatisfied / 100=Satisfied)**

1. **(Depends on the first option of question 10)** Regarding the AI-powered medical education platforms currently offered by your institution, what specific functions are included, and what functions would you like to see added in the future? **[Single-choice matrix] ***

| **Item** | **Option** | |
| --- | --- | --- |
| Literature Translation and Interpretation | □Available | □Not available |
| Exam Question Analysis | □Available | □Not available |
| Virtual Simulation Platforms | □Available | □Not available |
| Knowledge Mapping | □Available | □Not available |
| Intelligent Emotional Support | □Available | □Not available |
| Basic Laboratory Support | □Available | □Not available |
| Clinical Trial Assistance | □Available | □Not available |
| Frontier Knowledge Navigation | □Available | □Not available |

1. **(Depends on the second and third options of question 10)** If an AI-powered medical education platform is launched in the future, what aspects would you expect most? Please rank the following items in order of importance. **[Multiple-choice ranking question]***

□Literature Translation and Interpretation

□Exam Question Analysis

□Virtual Simulation Platforms

□Knowledge Mapping

□Intelligent Emotional Support

□Basic Laboratory Support

□Clinical Trial Assistance

□Frontier Knowledge Navigation

□Other (Please fill in the blank: __________ )

_________________________________

If you wish to receive updates on this research, you may provide your contact information below.

1. mail: ______________________________**(Optional)**

To inquire about research progress or for any questions, you may contact us through the following channel:

**E-mail: (anonymous)**

Thank you for participating in this survey!
