# Supplementary material for: Utilization of AI Among Medical Students and Development of AI Education Platforms in Medical Institutions: Cross-Sectional Study
Source: JMIR Hum Factors. 2026 Jan 8;13:e81652. doi: 10.2196/81652 (PMC12782625; doi:10.2196/81652)
Supplement: Checklist 1 [file humanfactors-v13-e81652-s002.pdf]

Checklist for Reporting Results of Internet E-Surveys (CHERRIES)

Item Category	Checklist Item	Explanation
Design	Cross-sectional survey	An anonymous online questionnaire was administered to assess AI usage in learning, student feedback on AI-powered medical education platforms, and expected functionalities among medical students from different disciplines, different educational stages and different academic programs in Shanghai, China.
IRB (Institutional Review Board) Approval and Informed	IRB approval	Study was determined to be Exempt by IRB
Consent process	Informed consent	Survey was conducted through an online data collection service (Questionnaire Star). Informed consent was provided in the cover page and participants had to click agree before proceeding to the survey.
	Data Protection	Surveys were anonymous in order to protect unauthorize access. Data access was restricted to research team members, and all data were used solely for analysis and reporting within this study.
Development and testing		Based on the study objectives and preliminary findings. Validity was established via preliminary testing.
Recruitment process		

and description of the sample having access to the questionnaire	The survey was conducted through open recruitment. Participants obtained the survey link via WeChat groups or learned about the study through offline poster advertisements at medical schools.
Survey administration	The survey was available through Questionnaire Star (a website); responses were automatically captured
Context	Participants received the survey link through WeChat, and the link was also available on the Questionnaire Star platform.
Mandatory	The survey was voluntary and participants could stop at anytime.
Incentives	No incentives were offered
Time/Date	March 1, 2025- May 31, 2025
Randomization of items or questionnaires	No
Adaptive questioning	If participants do not self-identify as currently undergoing medical specialty education, the first question will terminate their participation in this survey.
Number of Items Per page	3-8 questions per page
Number of screens	2 screens
Completeness check	Yes; Questionnaire Star alerts participant of completion. However, participants were allowed to skip questions or to stop at any time.

Review step	Respondents could go to previous page to review their answers
Response rates	
Unique site visitor	Did not provide participation rate
Participation rate	440/521 84.4%
Completion rate	428/440 97.3%
Preventing multiple entries	Cookies and IP address was not used to track individuals
Analysis	
Were only completed questionnaires analyzed?	No
Were questionnaires terminated early also analyzed?	No
Questionnaires submitted with an atypical timestamp	No
Statistical correction	All data were processed and analyzed using Microsoft Excel and SPSS.
